# The effect of cadet resilience on self-efficacy and professional achievement: verification of the moderated mediating effect of vocational calling

**DOI:** 10.3389/fpsyg.2023.1330969

**Published:** 2024-01-08

**Authors:** Olga Navickienė, Aidas Vasilis Vasiliauskas

**Affiliations:** Logistics and Defense Technology Management Research Group, General Jonas Zemaitis Military Academy of Lithuania, Vilnius, Lithuania

**Keywords:** cadets, self-efficacy, resilience, professional achievement, vocational calling, moderated mediating effect

## Abstract

**Background:**

The primary objective of this study was to empirically examine the influence of cadets’ resilience on their professional achievement within the unique context of a Military Academy. In doing so, the study sought to delineate the role of self-efficacy as a key mediator in the intricate relationship between the resilience of cadets and their professional achievements. The main focus of this study was to clarify the causal and effect relationships between the psychology and behavior mechanisms of the cadets. This was achieved through rigorous scrutiny of the moderated mediating effect of vocational calling within the multifaceted relationship involving cadets’ resilience, self-efficacy, and professional achievement.

**Methods:**

The study’s participant pool consisted of 121 individuals, comprising cadets in their third and fourth years of study, all of whom aspired to attain the rank of officer within the Military Academy. To rigorously investigate the hypotheses presented, a series of causal relationships among the four core variables were evaluated using a robust regression analysis methodology. To facilitate this analysis, the PROCESS macro 3.5v, a Hayes-developed tool, was effectively used.

**Results:**

The findings of this study revealed several critical insights. First, vocational calling emerged as a potent moderating factor in shaping the relationship between cadets’ resilience and self-efficacy. Furthermore, it was demonstrated that vocational calling exerted a conditional influence on the impact of cadets’ resilience on their professional achievement, with self-efficacy serving as a crucial mediating mechanism in this relationship. In particular, the study affirmed that self-efficacy functioned as a comprehensive mediator, elucidating the pathway through which the resilience of the cadets ultimately influenced their professional achievements.

**Conclusion:**

The results of this research contribute significantly to enhancing our understanding of the intricate connection between the resilience levels exhibited by cadets and their corresponding professional achievements. Furthermore, these findings have valuable implications for the ongoing refinement of military education and training programs. They offer insights that could inform the development of more effective testing and selection protocols for military personnel, ultimately benefiting the armed forces in their pursuit of excellence.

## 1 Introduction

The selection and training of military personnel have assumed paramount significance in contemporary times, as underscored in recent scholarly literature (Haralambie, 2016; Rodden-Aubut and Tracey, 2022). Indeed, the effective performance of soldiers has perennially represented a major challenge for armed forces worldwide (Sellman et al., 2010). However, the landscape of military effectiveness is evolving, with increasing emphasis on individual contributions of soldiers (Shamir and Ben-Ari, 2018). This transition towards recognizing the key role of individual actions within the military domain highlights the need for more comprehensive and nuanced research efforts. The evolving dynamics of warfare, with contemporary operational scenarios often demanding swift, adaptable, and strategic responses from soldiers, require a more profound understanding of the factors and psychological attributes that underpin military effectiveness (Goodwin et al., 2018; Bekesiene, 2023; Covington et al., 2024). Given this evolving landscape, it is evident that contemporary research in this domain is instrumental not only in shaping the selection and training processes but also in enhancing overall military performance and readiness in an era marked by dynamic challenges and shifting paradigms (Britt et al., 2013).

In the sphere of military professionalism, encompassing soldiers and officers alike, adherence to rigorous standards is not just an expectation, but a fundamental requirement (Nindl et al., 2018). These exacting standards are essential in equipping military personnel to navigate the myriad of challenging scenarios they may encounter. In this context, the concept of self-efficacy assumes a key role, as it refers to an individual’s firm belief in their ability to competently execute tasks, overcome impediments, and succeed in specified circumstances (Boe et al., 2018). Self-efficacy, grounded in an individual’s self-perception of their own capabilities, serves as a cornerstone in the soldiers’ capacity to confront and effectively manage unforeseen and demanding situations. This psychological attribute fosters resilience, adaptability, and resourcefulness among military professionals, allowing them to rise to the occasion and overcome adversities, on the battlefield or in complex operational environments (Coughlin, 2018). Recognizing the important role of self-efficacy within military contexts is essential not only to understand the psychology of soldiers, but also to optimize training, leadership, and support systems to fortify their ability to perform under the most challenging of circumstances. This understanding is an indispensable facet in ensuring the continued excellence and readiness of military forces in the face of evolving challenges and exigencies (Djourova et al., 2020; Bekesiene et al., 2022, 2023; Kanapeckaitė et al., 2022).

Soldiers and officers who exhibit elevated levels of self-efficacy are notably predisposed to undertaking and excelling in challenging tasks, demonstrating unwavering persistence in the face of adversity, and displaying remarkable adaptability in the ever-evolving landscape of military operations (Myrseth et al., 2018). This psychological attribute, self-efficacy, fundamentally influences the approach of these individuals toward tasks, imbuing them with unwavering confidence and the unshakeable belief in their capacity to achieve success, even in the most demanding circumstances. This optimistic and self-assured mindset exerts a profound and affirmative impact on individuals’ performance within military contexts. Self-efficacious military professionals are notably inclined to set ambitious goals and exhibit the requisite determination and exertion to achieve them. Consequently, they are shown to be instrumental in not only achieving individual success but also enhancing the collective effectiveness of military teams. In essence, the development and cultivation of high levels of self-efficacy among military personnel emerge as a crucial and multifaceted element in the acquisition of specific military skills and capabilities (Johansen et al., 2014; Boe et al., 2018). These attributes not only enhance individual proficiency, but also serve as key drivers in overall preparedness, performance, and mission success of military units, highlighting the critical role of self-efficacy within the military domain.

Within the military context, the demand for rapid decision-making and effective problem solving in high-stress situations is a recurring and formidable challenge encountered by military professionals (Myrseth et al., 2018; Smaliukienė et al., 2023). In this crucible of pressure, the presence of strong self-efficacy emerges as a crucial psychological resource. Self-efficacious military professionals have an unwavering belief in their ability not only to confront but also to overcome the multifaceted challenges that high-stress scenarios present. This enhanced sense of self-efficacy contributes significantly to their stress management abilities by instilling the confidence needed to navigate adversity, ultimately reducing anxiety levels, improving focus, and improving their ability to make informed decisions under duress. Furthermore, resilience is an indispensable trait within military circles, encapsulating the ability to rebound from setbacks, adapt to new and dynamic situations, and maintain overall well-being (Harms et al., 2018; Dimas et al., 2021). Individuals with high self-efficacy exhibit a remarkable perspective in the face of adversity, characterizing setbacks as transient obstacles that can be overcome with unwavering effort and unwavering determination. This mindset empowers them not only to bounce back from failures but also to distil valuable lessons from their experiences. Armed with this psychological resource, they persist in their pursuit of goals with resolute determination, bolstering their ability to contribute to mission success and overall well-being within the military environment (Nindl et al., 2018). It can also be said that the interplay of self-efficacy, stress management, and resilience within the military context unveils a nexus of psychological attributes that significantly influence the performance, well-being, and adaptability of military professionals. These qualities are fundamental to navigate high stress and formulating an unwavering and resolute approach to adversity and challenges encountered in military operations (Blacker et al., 2019; Flood and Keegan, 2022).

In the realms of occupational psychology and organizational behavior, contemporary vocational calling is recognized as having profound social significance. Vocational callings represent more than mere career choices; militaries are deeply rooted in personal passions, values, and a sense of purpose (Hall and Chandler, 2005). Contemporary vocational callings are intrinsically linked to personal fulfilment and identity (Ahn et al., 2017; Duffy et al., 2018). When people find vocations that align with their true calling, they experience a deep sense of purpose and satisfaction (Hall and Chandler, 2005; Duffy and Dik, 2013). This alignment with one’s calling leads to a stronger sense of identity, as individuals see their work as an integral part of who they are. Experts in occupational psychology have emphasized the importance of identifying and nurturing these callings to achieve higher levels of personal well-being (Bimrose and Hearne, 2012; Slemp et al., 2015). The notion of vocational calling extends to the pursuit of balance and well-being in contemporary society. Experts in occupational psychology stress the importance of a work-life balance (Akanji et al., 2020). Vocational callings that prioritize personal growth, mental health, and flexibility help people achieve a healthier work-life balance. This, in turn, contributes to improved mental health and overall well-being (Redekopp and Huston, 2020).

In pursuit of comprehending the intricate interplay among vocational calling, resilience, self-efficacy, and their impact on cadets’ military performance, this study was fundamentally designed to investigate the moderated mediation effects of self-efficacy in the relationship between resilience and military performance. Research sought to unravel the nuanced dynamics that underpin how vocational calling influences resilience, and, in turn, how self-efficacy acts as a mediator, channelling these influences to affect the ultimate outcome of military performance. To achieve this, the study used a methodological framework that encompasses a multifaceted analysis of these constructs, allowing exploration of interconnectedness and the potential moderating role of vocational calling. This research represents a critical step towards a more comprehensive understanding of the intricate mechanisms that drive professional success and well-being within military cadets, shedding light on the pivotal role that vocational calling, resilience, and self-efficacy play in shaping their performance and outcomes in the military domain.

In the realm of academic research, prior investigations have predominantly focused on the evaluation of students’ beliefs and attitudes pertaining to their capacity to attain academic excellence (Caprara et al., 2011; Zimmerman, 2013; Hayat et al., 2020; Bekesiene, 2023). These studies have frequently employed self-efficacy as a mediating factor to understand the relationship between such beliefs and actual academic performance within civilian educational institutions (Yu et al., 2016; Alyami et al., 2017; Doménech-Betoret et al., 2017; Li et al., 2022). However, the literature offers a limited scope when it comes to unravelling the implications of vocational calling on the intricacies of military performance. This lack of information underscores the need for a comprehensive exploration of the multifaceted dynamics that connect resilience, self-efficacy, and collective influence within the military context. In response to this gap, the main objective of this study was to conduct a rigorous examination of direct and indirect pathways that interconnect resilience and self-efficacy within the unique framework of military cadet performance. By exploring these pathways, this research sought to expand our understanding of how psychological attributes such as resilience and self-efficacy contribute to the multifaceted landscape of military success, offering valuable insights that can inform military training, education, and overall performance enhancement strategies.

## 2 Theoretical background

### 2.1 Resilience

Resilience in the context of militaries refers to the ability of military organizations and their personnel to withstand, adapt to, and recover from challenges, crises, and adversities while maintaining their operational effectiveness and mission readiness (Britt et al., 2013). It encompasses various aspects of preparation, response, and recovery, both at the organizational and individual levels (Carlson et al., 2012). Additionally, resilience plays an important role in underpinning the vocational calling, influencing individuals’ ability to pursue and sustain a career path driven by a deep sense of purpose and passion (Kossek and Perrigino, 2016; Salisu et al., 2020). Therefore, in the pursuit of a vocational calling, militaries often face challenges, setbacks, and obstacles. Thus, resilience enables them to face these challenges with determination and persistence, allowing them to stay committed to their chosen path even in the face of difficulties (Skinner and Pitzer, 2012).

In particular, the vocational calling is often characterized by strong internal motivation and a deep sense of purpose (Hall and Chandler, 2005; Woodruff et al., 2006; Mileham, 2010; Osterberg et al., 2020). There resilience can help militaries maintain their motivation over the long term. During service time, when soldiers face obstacles or experience failures, their resilience allows them to adapt, learn from their experiences, and continue working toward their vocational goals (Snider and Watkins, 2000). Moreover, vocational callings can evolve over time as individuals grow and develop, and resilience is essential for adapting to these changes, whether it involves refining one’s career goals, exploring new avenues, or transitioning into different roles while still staying true to one’s calling.

In addition, resilience fosters self-efficacy, which is the belief in one’s ability to overcome challenges and achieve goals (Howe et al., 2012; Cassidy, 2015). With a strong sense of self-efficacy, militaries pursuing a vocational calling are more likely to persevere and make the necessary efforts to succeed in their chosen field.

Military resilience can be stated to be a multifaceted concept that encompasses a wide range of skills, strategies, and capabilities that aim to ensure that military forces can continue to fulfil their roles and missions even in the face of adversity and uncertainty (Wood et al., 2019). It is an essential aspect of modern military planning and preparedness. Overall, for military personnel, resilience serves as a critical foundation for vocational callings by providing individuals with the psychological and emotional resources needed to navigate the challenges, uncertainties, and personal growth that come with following a deeply meaningful career path. It helps them persist, adapt, and thrive in their chosen vocations, allowing them to fulfil their sense of purpose and passion.

### 2.2 Self-efficacy

Self-efficacy, as defined by Bandura (1989), refers to the belief in one’s ability to perform a specific task. It influences an individual’s goal-seeking behavior by determining the level of intensity with which they pursue a particular objective. It is important to note that self-esteem, which involves self-respect, differs from self-efficacy, as the latter is rooted in the belief in one’s own capabilities (Gardner and Pierce, 1998).

Bandura (1989) emphasized the significance of the social environment, human cognition, and behavioral abilities in learning and development through his social cognitive theory. He recognized self-efficacy as a more powerful motivator for purpose-seeking behavior than self-esteem or self-satisfaction. Over time, Bandura (2005) developed his social cognitive theory into a comprehensive model where behavioral, cognitive, and environmental factors interact and influence each other, giving rise to new psychological dynamics.

The causal relationship between one’s perception of their abilities and their performance in a specific role is influenced and structured by self-awareness, along with social and psychological conditions (Harrison et al., 1997; Dybowski et al., 2017). Motivated individuals possess the confidence that enables them not only to excel in specific behaviors, but also to perform effectively in diverse tasks and unconventional situations (Gardner and Pierce, 1998; Qiu et al., 2023). Self-efficacy does not just impact current job performance; it also plays a significant role in influencing future organizational behavior. As a result, self-efficacy is considered a psychological variable that predicts a military personnel’s performance in the workplace and their organizational behavior (Smaliukienė et al., 2023).

### 2.3 Vocational calling

Contemporary vocational vocations are ascribed profound social significance by experts in the fields of occupational psychology and organizational behavior (De Vos et al., 2020). This phenomenon is understood as a manifestation of altruistic inclinations, wherein individuals are driven by an inherent desire to contribute to the welfare of others and the broader societal fabric, rather than merely pursuing self-interest or personal gain (Kossek et al., 2021). This altruistic perspective on vocational callings underscores the evolving understanding of work as a means of not only personal fulfilment but also a noble endeavour to enhance the collective well-being of humanity. This shift in perspective sheds light on the complex interplay between individual motivations and the broader societal context within which vocational choices are made, highlighting the importance of social and ethical dimensions in the contemporary workforce (Fassinger, 2008; Afsar et al., 2019a).

In more precise terminology, the concept of vocation is inherently linked to a set of professional values, wherein the individual derives profound gratification and a profound sense of meaning from his work. This sense of fulfilment transcends mere material gain or the traditional markers of social status, instead focusing on the intrinsic rewards of their vocational pursuit (Quigley and Tymon, 2006). Individuals who are in tune with their vocational calling tend to engage in self-directed learning and exhibit innovative behavior, often grounded in the psychological ownership they feel toward their work. This ownership mindset fosters an ongoing process of skill and knowledge development, driven by a strong internal motivation to improve adaptability and contribute meaningfully to their chosen field (Ahn et al., 2017). This interplay between vocation, self-directed learning, and innovative behavior underlines the importance of intrinsic motivation and personal growth in the vocational domain. It reflects a deep-seated commitment to continuous self-improvement, as individuals autonomously acquire the necessary competencies to excel in their roles and adapt to the dynamic demands of their profession (Haynie and Shepherd, 2011). The vocational calling, therefore, not only transcends extrinsic rewards, but also serves as a catalyst for individual growth and professional evolution. This holistic perspective underscores the intricate nature of the relationship between vocational satisfaction, self-directed learning, and the cultivation of skills required for sustained success and adaptability in the contemporary workplace (Hall and Chandler, 2005).

Building upon the framework of self-determination theory (SDT), as elucidated by Lee (2016), the concept of a “sense of calling” emerges as a pivotal psychological mechanism with significant implications on the determination of one’s approach to tasks and preferred production methods. SDT, a prominent theory in the realm of motivation and personality, posits that individuals have innate psychological needs for autonomy, competence, and relatedness. Within this theoretical framework, the sense of calling plays a critical role. A sense of calling, as defined by Lee (2016), becomes a lens through which individuals interpret their professional endeavours and work-related tasks. It serves as an internal compass that guides people in their quest for autonomy and competence, enabling them to engage in tasks with a heightened sense of purpose and intrinsic motivation. This sense of calling shapes not only the choices individuals make in terms of their career paths, but also how they approach and execute the tasks within those paths. Additionally, a sense of calling is intricately tied to the way individuals choose to execute their work. It influences their preferred production methods, as individuals driven by a strong sense of calling often seek innovative, meaningful, and purpose-driven approaches to task completion. This can result in a more engaged and proactive work style, as well as a proclivity to find and create new, more effective methods to achieve their professional objectives. All in all, Lee’s (2016) incorporation of the concept of a sense of calling within the framework of SDT underscores the profound impact that this intrinsic motivational force has on an individual’s vocational choices, approach to tasks, and preferred production methods. Highlight the interplay between intrinsic motivation, psychological needs, and practical aspects of professional work, shedding light on the complex dynamics that underlie human behavior at work.

The latest work of Tomprou and Bankins (2019), adopting a positive psychology perspective, offers a nuanced interpretation of vocational calling. They conceive it as a profound willingness to engage in diverse and multifaceted roles, both within and beyond the boundaries of the traditional working environment. Their perspective aligns with the broader tenets of positive psychology, which accentuate the significance of personal strengths, well-being, and the realization of one’s full potential.

Within this positive psychology framework, vocational calling transcends mere job-related tasks and responsibilities (Dumas and Sanchez-Burks, 2015). It encompasses a broader spectrum of roles that individuals willingly assume, driven by an inner calling to make a meaningful and multifaceted contribution to society. This interpretation emphasizes the holistic nature of vocational calling and its intrinsic link to personal growth, self-fulfilment, and the cultivation of a well-rounded sense of self (Duffy and Dik, 2013; Ji and Yoon, 2021).

Moreover, the recent surge of studies on vocational calling has been particularly intriguing due to their recognition of a division between the presence of a calling and the active search for a calling (Wells, 2012). This distinction underscores the dynamic interaction between social and psychological variables. The presence of a calling suggests an individual’s alignment with a well-defined vocational path, where they experience a profound sense of purpose and fulfilment (Murphy and Kreiner, 2020). In contrast, the search for a calling reflects a more exploratory phase, where individuals are in the process of discovering their true vocational identity.

This division not only sheds light on the evolving nature of vocational calling, but also highlights the intricate interrelationship between personal psychology and the social context in which these callings are nurtured and realized (Duffy and Dik, 2013). It underscores the role of societal influences, personal exploration, and self-discovery in the development of vocational callings, contributing to a deeper understanding of the complex dynamics that underlie this phenomenon.

### 2.4 Professional achievement in the military

Professional achievement within the military is a multifaceted and profound concept that reflects the dedication, competence, leadership, and contributions of service members within the armed forces (Johansen et al., 2014; Šimanauskienė et al., 2021). It signifies not only individual success, but also the collective strength of the military as an institution (Angstrom and Haldén, 2019). One of the most visible and universally recognized indicators of professional achievement in the military is the attainment of higher ranks and positions. Promotion through the ranks signifies an individual’s competence, experience, leadership skills, and contributions to the organization (Townley, 2019). It is a clear testament to the trust and responsibility placed in the service member. Advancement often requires a combination of experience, education, and successful performance, and is a significant aspiration for many military personnel (Lacerenza et al., 2018).

## 3 Research method

### 3.1 Research models and hypotheses

**Figure 1:**
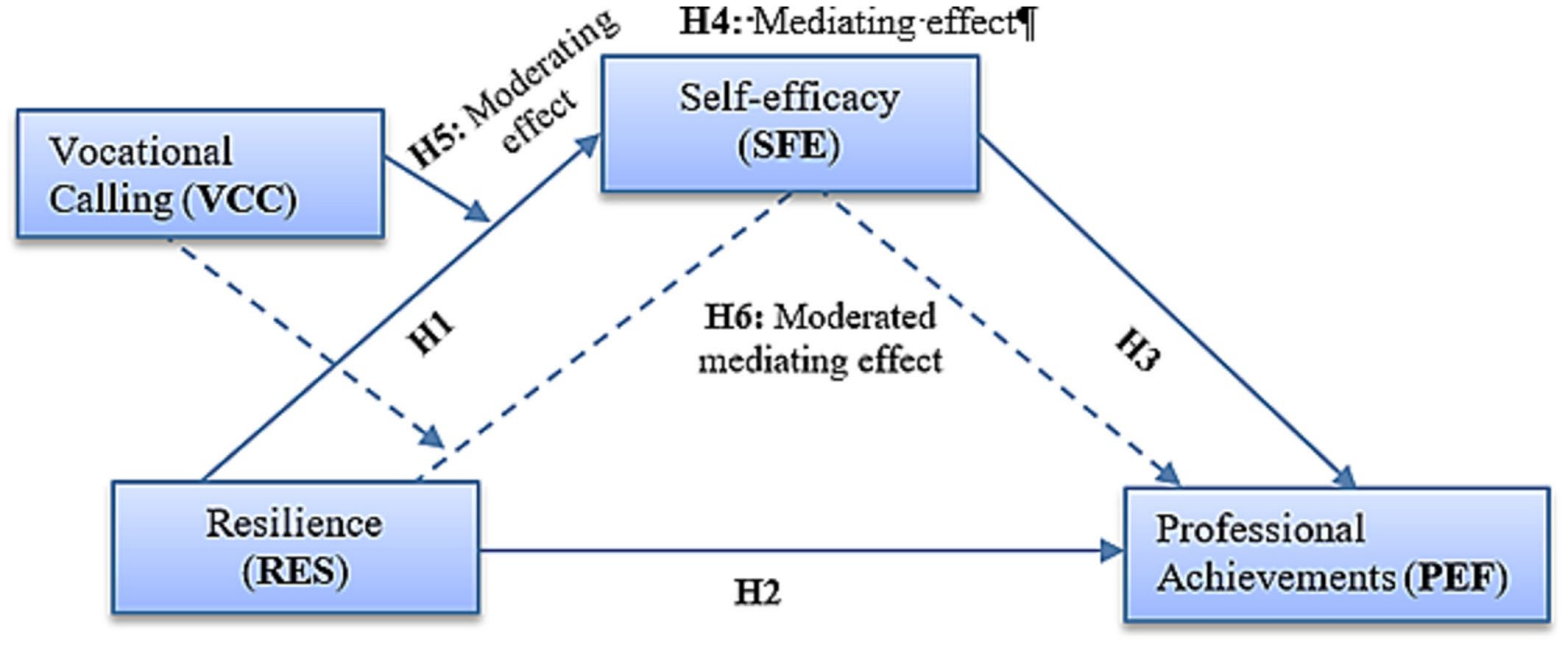
The hypothesized theoretical model delineates both the direct and indirect effects of cadets’ resilience (RES). Specifically, the direct effects are specified with regard to self-efficacy (Hypothesis H1) and professional achievements (Hypothesis H2). Additionally, the model encompasses the examination of the indirect effects (Hypothesis H4) of cadets’ resilience on professional achievement (PEF) through its impact on self-efficacy (SFE). Furthermore, Hypothesis H5 aims to assess the indirect effects of vocational calling (VCC) in its role as a moderator within the relationship between cadets’ resilience and self-efficacy. Finally, Hypothesis H6 is formulated to test the moderated mediating effect of vocational calling on self-efficacy in the association between cadets’ resilience and professional achievement (see dashed lines).

This study meticulously constructed a comprehensive framework of interrelated variables, grounded in the theoretical underpinnings of cadet resilience, self-efficacy, vocational calling, and professional achievements. This theoretical foundation served as the basis for the formulation of a research model, visually represented in Figure 1, which was designed to empirically investigate the impact of the resilience of the cadets on both self-efficacy and the professional achievements of future military officers.

Furthermore, the study delved into the nuanced role of vocational calling as a potential moderating mediator within the complex relationship between cadets’ resilience and self-efficacy. This model aimed to provide a systematic and data-driven exploration of these intricate dynamics, shedding light on the underlying mechanisms that shape the professional development of cadets within the context of a military academy.

### 3.2 Causality between resilience, self-efficacy, and professional achievement of cadets

The military environment is a crucible that places great demands on the physical, mental, and emotional fortitude of the cadets. Achieving professional success and effectiveness within the military requires a unique blend of attributes, including resilience and self-efficacy (McLarnon et al., 2021).

Resilience, the ability to adapt and bounce back from adversity, forms the bedrock of a cadet’s journey towards professional achievement. Cadets often face formidable challenges, ranging from demanding training routines to high-pressure scenarios in the field. Resilience equips them to withstand setbacks, persevere in the face of adversity, and emerge stronger from the experience. As experts in the fields of psychology and military science assert, resilience is a key determinant of success within the military (Nindl et al., 2018; Jones et al., 2022).

Self-efficacy, grounded in an individual’s belief in their ability to execute tasks successfully, assumes a central role in a cadet’s journey. High self-efficacy engenders confidence and optimism, leading cadets to approach challenges with a sense of competence. Cadets with strong self-efficacy tend to set ambitious goals, persevere through adversity, and exert the effort required for success. In the military context, self-efficacy is essential to make quick decisions, manage stress, and navigate complex tasks (Bergman et al., 2019; Dwyer, 2019; Likhomanov et al., 2020).

The causality between resilience and self-efficacy is a reciprocal relationship. Resilience, developed through the experience of facing and overcoming challenges, fosters self-efficacy. The more cadets overcome adversity, the more they come to believe in their ability to deal with future challenges. This relationship is well documented in research within military and psychological fields (Bandura, 1997; Smith et al., 2018). In essence, resilience acts as the crucible in which self-efficacy is forged, and self-efficacy, in turn, bolsters resilience.

Professional achievement within the military is the result of the resilient and self-efficacious drive of cadets. Resilience enables them to endure rigorous training, recover from setbacks, and adapt to ever-changing military scenarios. Self-efficacy empowers them to lead with confidence, make decisive decisions, and excel in the field. Consequently, cadets with high levels of resilience and self-efficacy tend to achieve promotions, excel in leadership roles, and contribute significantly to mission success (Fosse et al., 2015). The causality between cadet resilience, self-efficacy, and professional achievement underscores an interdependent relationship (Gilson et al., 2016). Resilience and self-efficacy reinforce each other, propelling cadets toward their goals and fostering adaptability, determination, and a robust capacity to thrive in the military domain (Davis et al., 2019; Bekesiene, 2023).

Drawing on the extensive body of existing literature and well-established theoretical assumptions, this study hypothesized direct pathways within the proposed model, as depicted in Figure 1. Specifically, research hypotheses posit a direct influence that stems from the resilience of cadets to self-efficacy and professional achievement. These postulations are grounded in cumulative knowledge and theoretical frameworks that underscore the importance of resilience as a precursor to self-efficacy and, subsequently, professional accomplishments.

Direct effect of resilience on self-efficacy:

*Hypothesis 1 (H1)*. The resilience of the cadets will exert a positive influence on their self-efficacy.

Direct effect of resilience on cadets’ professional achievements:

*Hypothesis 2 (H2)*. The resilience of the cadets will have a positive effect on their professional achievement.

Direct effect of self-efficacy on professional achievement:

*Hypothesis 3 (H3)*. Self-efficacy will have a positive effect on professional achievement.

### 3.3 The self-efficacy as mediator

Self-efficacy, a concept rooted in the cognitive theory of social learning, has emerged as a prominent mediator in various domains, serving as a pivotal psychological bridge between one’s beliefs and their achievements. Self-efficacy, as conceptualized by renowned psychologist Albert Bandura, refers to an individual’s belief in their ability to execute specific tasks successfully. This belief extends beyond mere confidence; it encompasses a deep-rooted conviction in one’s capability to confront challenges, persevere through adversity, and achieve desired outcomes. Bandura’s social cognitive theory posits that self-efficacy plays a pivotal role in shaping human behavior and performance (Bandura, 1994).

In the realm of education, self-efficacy mediates the relationship between students’ beliefs and their academic achievements. Students who believe in their ability to learn and excel in their studies are more likely to set challenging goals, engage in active learning, and persist through difficulties. This self-belief not only influences their motivation, but also affects their approach to learning. Research has consistently shown that self-efficacy is a potent mediator of academic success (Zimmerman, 2000; Caprara et al., 2011).

In the domain of career success, self-efficacy is instrumental in mediating the relationship between an individual’s beliefs and their professional achievements (Eccles and Wigfield, 2020; Eliyan et al., 2020; Bekesiene, 2023). Those who have a strong sense of self-efficacy are more likely to set ambitious career goals, persist through setbacks, and demonstrate the determination needed for career advancement. Research in the field of organizational psychology highlights self-efficacy as a predictor of job performance and career success (Stajkovic and Luthans, 1998).

The self-efficacy mediating mechanism operates through a cascade of psychological processes. It influences goal setting, self-regulation, decision making, and resilience. Individuals with high self-efficacy tend to set more challenging goals, self-monitor their progress, make adaptive decisions, and bounce back from failures (Baron and Kenny, 1986; Hoyle, 2006; Baron et al., 2016). These processes collectively mediate the relationship between self-efficacy beliefs and outcomes. It can be stated that self-belief in one’s capabilities acts as a catalyst, propelling individuals towards their goals, and mediating the relationship between their beliefs and outcomes. Taking this into account, hypothesis H4 was formulated.

*Hypothesis 4 (H4)*. Self-efficacy will mediate between cadets’ resilience and professional achievements.

### 3.4 Moderating effect of vocational calling

Vocational calling, often referred to as a “calling” to one’s career, is a sociopsychological variable that has garnered significant attention in recent years. This concept delves into the intricate interplay between an individual’s career, personal identity, and societal contributions.

A vocational calling transcends the notion of a mere job or career; it encompasses a deep sense of purpose and personal fulfillment derived from one’s work. It is the profound belief that one’s professional path aligns with their core values, passions, and innate talents. Psychologically, it signifies the desire to contribute meaningfully to society through one’s work, often driven by a strong sense of responsibility and moral imperative (Duffy and Dik, 2013).

Vocational calling plays a pivotal role in shaping an individual’s self-concept and personal identity. When individuals perceive their work as a calling, it becomes an integral part of who they are. Their self-concept extends beyond the limits of their job description, reflecting a deeper connection between their personal identity and their vocational identity (Aulthouse et al., 2017). This interconnectedness fosters a sense of authenticity, self-esteem, and overall well-being.

The military profession demands unwavering resilience and self-efficacy from cadets as they navigate the complex challenges of training, leadership, and operational missions. However, a less explored dimension in this context is the impact of vocational calling.

Resilience, the ability to bounce back from adversity and adapt to challenging situations, is an essential trait for military cadets. Resilience enables them to withstand rigorous training, cope with stress, and emerge stronger from setbacks. It is a psychological resource that empowers cadets to thrive in the face of adversity, making it a critical determinant of success within the military (Harvey et al., 2018).

The hypothesis under consideration is that vocational calling plays a moderating role in the relationship between cadets’ resilience and self-efficacy. Vocational calling is expected to strengthen the connection between resilience and self-efficacy. Cadets who perceive their military service as a calling can draw additional motivation and a stronger belief in their abilities from this deep sense of purpose, amplifying the relationship between resilience and self-efficacy. In essence, vocational calling may serve as an additional source of empowerment, accentuating the psychological and emotional resources available to cadets.

Seco and Lopes (2013) identified the notable moderating and moderated mediating effects of vocational calling in the connection between authentic leadership and job commitment. In a separate study, Park et al. (2019) demonstrated that professional self-efficacy serves as a mediating factor between calling, job performance, and organizational civic behavior among salespeople in an insurance company. In particular, based on the studies mentioned, this research also emphasized the importance of a sense of vocational calling as a moderating factor in the relationship between calling and cadet professional achievement. In light of this, hypotheses H5 and H6 were formulated:

*Hypothesis 5 (H5)*. Vocational calling will play a moderating role in the relationship between cadet resilience and self-efficacy.

*Hypothesis 6 (H6)*. Vocational calling will moderate the mediating effect of self-efficacy in the relationship between resilience and the professional achievements of the cadets.

## 4 Research methodology

### 4.1 Design, place of study, and ethical aspects

The present study used a rigorous random sampling method to ensure the representative nature of the data. Data collection took place in February 2023 within the confines of the Lithuanian Military Academy (LMA). Using a digital format, self-reported questionnaires were administered to participants through the Google Forms platform.

Before entering the questionnaire, great care was taken to inform the cadets of the ethical principles governing this research. Emphasis was placed on the absolute guarantee of anonymity and the preservation of data confidentiality. It is imperative to emphasize that participation in the study was entirely voluntary and that no external incentives or rewards were offered to participants.

The sample under scrutiny consisted of 121 cadets, all of whom were currently enrolled in the 3rd and 4th courses at the LMA. Each participating cadet was presented with a comprehensive explanation of the study’s objectives and procedures. Additionally, informed consent was obtained from each student prior to their participation in the research. This process ensured that the cadets willingly and knowingly participated while their anonymity was vigilantly safeguarded throughout the research effort.

### 4.2 Measures

#### 4.2.1 Background factors

In the structured research questionnaire, a set of demographic variables was thoughtfully integrated, specifically encompassing gender, age, level of civilian education, and military experience. These variables were included to provide a comprehensive description of the background characteristics inherent in the study participants. This methodological approach allows for understanding the diverse profile of individuals participating in the research. The assessment of participants’ educational achievement level was conducted employing a three-point scale, with 1 denoting ‘secondary school’, 2 representing ‘professional,’ and 3 signifying ‘Other.’ Gender was categorized as a dichotomous variable and was coded as 1 for ‘male’ and 2 for ‘female.’ Military cadet’ experience was quantified using the following codes: 1 for ‘I have no experience’, 2 for ‘Volunteer in professional military service (PMIS)’, 3 for ‘National Defence Volunteer Force (NDVF)’, 4 for ‘Sagittarius Union’ and 5 for ‘Other’. Additionally, age was measured as a parametric variable on an interval scale, ensuring an accurate representation of participants’ age-related data.

#### 4.2.2 Self-efficacy scale

In accordance with the insights offered by self-efficacy theorists, specifically Urdan and Pajares (2006), the use of a universal or generalized scale to assess self-efficacy among cadets would not be appropriate. This position is based on the understanding that cadets should evaluate their own effectiveness, taking into account their unique experiences within the distinct milieu of military training and academic studies, as emphasized by Boe et al. (2018). To address this, a specialized questionnaire was used to gauge self-efficacy in domains relevant to the academic endeavours of the cadets at the military academy.

Although it is worth noting that Buch et al. (2015) had previously validated statements to assess military academic self-efficacy, these statements were adapted for the specific purpose of measuring cadets’ perceived competence within the context of the military academy. This adaptation ensured that the questions were tailored to the nuances of the cadets’ experiences.

The questionnaire featured seven items, each designed to elicit responses regarding cadet self-efficacy, measured on a 5-point scale. This scale ranged from 1, signifying ‘totally disagree,’ to 5, representing ‘totally agree.’ Following the guidance of Nunnally (1978) for scale reliability, it is notable that previous studies, such as those conducted by Buch et al. (2015) and Fosse et al. (2015), demonstrated a high degree of internal consistency for this self-efficacy scale. In both of these earlier studies, the Cronbach alpha coefficients exceeded 0.70, with values ranging from 0.83 to 0.89.

Furthermore, the current study maintains this respectable level of internal consistency, with the adapted self-efficacy scale showing a Cronbach alpha (α) of 0.892. This observation underscores the reliability and coherence of the scale in measuring the self-efficacy of the cadet within the specific context of the military academy, substantiating its suitability for rigorous scientific assessment.

#### 4.2.3 Professional achievement evaluation

Cadets’ professional achievement (PEF) was assessed through an instructor-rated evaluation system. This evaluation system is widely recognized and accepted by military training instructors, representing an effective measure of the improvement of cadets’ enhancement in military competencies. The assessment process involves the use of a 5-point Likert-type scale, where 1 signifies ‘below average,’ 2 corresponds to ‘slightly below average’, 3 denotes ‘average’, 4 represents ‘slightly above average’, and 5 signifies ‘above average’.

The evaluation covers a comprehensive range of military skills and attributes, encompassing 10 distinct domains: basics of first aid, preparation of equipment, recognition of topographic signs and object coordinates on the map, knowledge about weapons and shooting achievements, cooperation/communication, leadership, and coping. For each cadet, the responsible instructors assess their performance in each domain and assign a score based on the Likert scale.

To provide an overall assessment of the military capabilities of the cadets, the average score is computed across the 10 evaluated domains. This aggregated score offers a comprehensive overview of a cadet’s military proficiency. In particular, this approach ensures a well-rounded evaluation considering a multitude of crucial competencies. The military performance assessment tool exhibits a high degree of internal consistency, as indicated by a Cronbach alpha (α) coefficient of 0.803. This high level of internal consistency underscores the reliability and coherence of the assessment instrument, reinforcing its suitability to measure the achievement of the cadets in a scientifically rigorous manner.

### 4.3 Methods of statistical analysis

Before conducting statistical analyses, a sample size evaluation test was performed using the widely recognized software package G*Power version 3.1.9.4. (Faul et al., 2009). The objective was to assess the sample size necessary for the F tests in a linear multiple regression analysis, which involved three predictor variables. The specified criteria for this analysis included a significance level of 0.05, a desired statistical power of 0.95, and an anticipated effect size of 0.15. The outcome of the sample size evaluation test determined that a sample size of 119 would be required to achieve the desired statistical power of 0.95 for the specified analysis. Furthermore, a *post hoc* test was conducted to estimate the statistical power achieved. This *post hoc* analysis was performed with a significance level of 0.05, using a sample size of 121 from a valid dataset, and considering the same effect size of 0.15. The results of this *post hoc* analysis revealed that, even with a slightly reduced sample size of 117, a high statistical power of 0.954 could still be attained. These preanalysis procedures underscore the robustness of the statistical power achieved in the subsequent linear multiple regression analysis, even with a slightly smaller sample size than initially estimated, while maintaining the desired significance level and effect size.

The empirical research for this study was carried out using IBM SPSS 29v and IBM AMOS 29v statistical software packages. These software tools were instrumental in performing a series of essential statistical analyses. First, a frequency analysis was performed to systematically examine the demographic characteristics of the sample under scrutiny. This comprehensive analysis provided valuable insights into the composition of the study’s participants, allowing for a more nuanced understanding of the research context. Subsequently, a correlation analysis was performed to elucidate the interrelationships between the various measurement variables, serving as a crucial preliminary step before hypothesis testing (Tehseen et al., 2017). This analysis allowed for the assessment of the strength and direction of associations between the variables, helping in hypothesis formulation and model development (Fornell and Larcker, 1981). To ensure the validity of the measurement variables, a verification factor analysis was performed. This analytical step was crucial to confirm the reliability and validity of the measurement tools used in the research. In addition, the internal consistency of the measurement instruments was rigorously evaluated using the Cronbach alpha coefficient, enhancing the reliability of the data collected. The hypothesized relationships between the model constructs were assessed using SPSS AMOS version 29, and the coefficient weights were selected in alignment with the recommendations of previous researchers (Bekesiene et al., 2017a,b; Bekesiene et al., 2022; Smaliukienė et al., 2023). These scholars have advocated for a versatile methodology to assess the appropriateness of a theoretical model. Consequently, the goodness of fit for the models was evaluated using the following criteria: (1) the probability statistic of χ^2^ likelihood ratio, (2) the Tucker and Lewis Index (TLI), (3) the Comparative Fit Index (CFI), and (4) the Root Mean Square Error of Approximation (RMSEA), along with the corresponding confidence intervals (CI) (Hu and Bentler, 1999).

Finally, the six hypotheses of the study were rigorously tested through the application of SPSS PROCESS macro models 4 and 7, developed by Hayes (2017). These advanced statistical models were used to fulfil the overarching objectives of the study, facilitating a comprehensive analysis of the complex relationships and effects postulated in the research hypotheses. Furthermore, a bootstrapping analysis was performed involving 5,000 iterations and the acceptance of statistical significance was established at a 95% level for bias-corrected confidence intervals (95% CI). For the assessment of indirect relationships, it was deemed statistically significant if the value of zero was not included within the 95% bias-corrected CI. These analytical approaches align with the recommendations of scholars such as Hayes and Scharkow (2013) and Hair (2019).

## 5 Study results

### 5.1 Preliminary analyses of the data sample

The demographic characteristics of the sample indicate that 98 participants (81%) were male, while 23 (19%) were female. In terms of age distribution, 79 respondents (65.3%) were under the age of 19, 32 respondents (26.4%) were between 20 and 21 years old, 9 respondents (7.5%) fell within the age range of 22 to 24 years, and one respondent (0.8%) was over 25 years old. Regarding military experience, 71 participants (58.7%) had no prior military experience, 26 (21.5%) had volunteered at PMIMS, 19 (15.7%) had gained their experience in the Sagittarius union, a cadet (0.8%) mentioned that they had served in the NDVF, and 4 (3.3%) had other forms of military experience. Regarding education achievement, 120 participants (99.2%) were secondary school graduates, and only one (0.8%) had different educational credentials. Additional details are presented in Table 1.

**Table 1:** The demographic characteristics of study participants.

Demographic characteristics	M (±SD) or N (%)
Gender	
1: Male (%)	98 (81%)
2: Female (%)	23 (19%)
Age (M;±SD)	19.5 (±1.444)
Military experience (*N*; %)	
1: I have no experience	71(58.7%)
2: Volunteer at professional military service (PMIS)	26 (21.5%)
3: National defence volunteer force (NDVF)	1(0.8%)
4: Sagittarius union	19(15.7%)
5: Other	4(3.3%)
Education (*N*; %)	
1: Secondary	120 (99.2%)
2: Professional	0 (0%)
3: Other	1 (0.8%)

PMIMS, permanent mandatory initial military service; NDVF, national defence volunteer force.

Furthermore, Pearson’s correlation analysis was chosen to determine the correlation between resilience (RES), self-efficacy (SEF), vocational calling (VCC) and professional achievement (PEF). The results of the analysis conducted showed that the resilience of the cadets showed a significant and positive correlation between self-efficacy (RES&SEF, *r* = 0.568, *p* < 0.01), vocational calling (RES&VCC, *r* = 0.490, *p* < 0.01), and professional achievement (RES&PEF, *r* = 0.376, *p* < 0.01). Additionally, self-efficacy was identified as indicating positive and highly significant relationships with vocational calling (SEF&VCC, *r* = 0.691, *p* < 0.01). These results are presented in Table 2.

**Table 2:** The relationships between study variables.

Latent variables	Resilience	Self-efficacy	Vocational calling	Professional achievement
Resilience (RES)	1			
Self-efficacy (SEF)	0.568^**^	1		
Vocational calling (VCC)	0.490^**^	0.691^**^	1	
Professional achievement (PEF)	0.376^**^	0.463^**^	0.346^**^	1

** Pearson correlation significance at the 0.01 level (2-tailed).

### 5.2 Analysis of latent factors and verification of reliability

A verification analysis was conducted to verify the validity and suitability of each variable presented in this study (Table 3). The fit of the model for this was evaluated using the significance probability of χ2 test (normed fit index, *χ*^2^/DF = 1.675, *p* < 0.001), Tucker-Lewis index (TLI = 0.943); Confirmatory factor index (CFI = 0.965); Root mean square error of approximation (RMSEA = 0.075).

**Table 3:** Confirmatory factor analysis and reliability analysis of the whole composite concept.

Latent variables	Factor	λ	α	CR	AVE
Resilience (RES) 5	I am able to adapt when changes occur	0.752	0.838	0.888	0.616
	I can overcome anything	0.714			
	I stay focused and think clearly in stressful situations	0.757			
	Failures do not break my resolve so easily	0.856			
	I consider myself a strong person who can overcome life’s challenges and difficulties	0.834			
Self-efficacy (SEF) 6	I am a person who can graduate from General Jonas Žemaitis Lithuanian Military Academy (hereinafter - LKA)	0.855	0.892	0.916	0.646
	I will be able to mobilize and find the necessary strength to do the hard work related to my studies at LKA	0.851			
	I will be able to endure the most difficult moments of studying at LKA	0.822			
	I will be able to finish LKA with higher grades than my colleagues	0.758			
	I will achieve results that I can be proud of	0.785			
	After graduation, I intend to be an officer	0.743			
Vocational calling (VCC) 10	I like being an officer	0.852	0.946	0.953	0.671
	I think it’s fun to serve in the military	0.735			
	The military profession inspires me	0.888			
	I feel called to serve in the army	0.818			
	I find the service of an officer satisfying	0.803			
	I see opportunities to realize myself	0.829			
	The profession of an officer will help me to constantly deepen my knowledge and improve	0.866			
	Military service will help me improve my personal qualities	0.774			
	The activities of an officer correspond to my personal values	0.789			
	I am satisfied with my decision to pursue the military profession	0.826			
Professional achievement	Compile a detailed fire card	0.797	0.803	0.914	0.604
	Know the non-sound control signals	0.766			
	Prepare the equipment	0.782			
	Recognize topographic signs	0.773			
	Set object coordinates on the map	0.774			
	Weapons and shooting	0.793			

Chi-square (CMIN) = 962.867; degrees of freedom (DF) = 575; normed fit index (CMIN/DF) = 1.675; Tucker-lewis index (TLI) = 0.943; confirmatory factor index (CFI) = 0.965; root mean square error of approximation (RMSEA) = 0.075.

The central feasibility of the composition concept has been rigorously substantiated by empirical investigation, with a confirmed standard value of 0.5. Furthermore, its conceptual reliability, a critical measure of its consistency and robustness, has been established at an impressive level of 0.7. Additionally, the average variance extracted (AVE) for this concept stands at a commendable value of 0.5, exceeding the standard benchmarks, thus affirming its significant presence in the studied domain. These findings underline the scientific validity and strength of the composition concept within the context of the examined research framework. The measurement model used in this study is well regarded within the scientific community for its robustness and appropriateness. The reliability of the measurement model is substantiated by Cronbach’s alpha coefficients, with all variables consistently achieving values of 0.6 or higher, as established in previous research (Hair et al., 2014). This elevated level of internal consistency attests to the trustworthiness and dependability of the measurement model, thus reinforcing confidence in the data collected and the subsequent analyses undertaken in the study.

### 5.3 Hypotheses testing results

The analytical procedures were conducted with the help of advanced statistical software tools, specifically IBM AMOS version 29 and IBM SPSS version 29. To evaluate the postulated relationships among constructs within the specified models, the Confirmatory Factor Analysis (CFA) was employed. The use of CFA allowed for a rigorous examination of the theoretical framework and its associated constructs.

Furthermore, the hypotheses of the theorized model were evaluated using PROCESS macro version 3.5. This analytical tool facilitated a comprehensive examination of the intricate interplay and mediation effects between variables, allowing a deeper understanding of the underlying processes outlined in the research hypotheses. Such meticulous data analysis techniques enhance the robustness and precision of the research findings, aligning with contemporary scientific standards.

### 5.4 Hypothesis confirmation

In order to ascertain the potential moderating influence of vocational calling on the mediating mechanism of self-efficacy within the context of the relationship between cadets’ resilience and their innovative behavior, an advanced statistical approach was employed. Specifically, the PROCESS version 3.5 macro, Model 7, was used to perform this analysis.

To ensure the robustness of the results, a bootstrapping procedure involving 5,000 resamples was meticulously specified. This resampling method enhances the reliability of the estimated effects and their associated confidence intervals. Additionally, a confidence interval set at 95% was chosen, serving as a critical metric to determine the significance of the observed moderating effect. By adhering to these rigorous analytical techniques, a comprehensive evaluation of the moderating role of vocational calling in the mediating relationship between cadets’ resilience and innovative behavior was achieved.

First, following the analysis of the resilience of the cadets as an independent variable and its influence on self-efficacy as a dependent variable, it was revealed that hypothesis H1 is substantiated, indicating that the resilience of the cadets exerts a positive effect on self-efficacy (RES → SEF, for H1: β = 0.189, *p* < 0.01, see Model 1, Table 4).

**Table 4:** Causal relationships between the concept of theoretical model structure.

Predictors	(β)	SE	*t*	*p*	LLCI	ULCI
Model 1 (outcome variable: Self-efficacy (SEF))						
Constant	4.161	0.028	147.823	0.000	4.106	4.217
RES → SEF	0.189	0.059	3.227	0.002	0.073	0.305
VCC → SEF	0.556	0.069	8.028	0.000	0.419	0.693
RES × VCC → SEF	−0.217	0.096	−2.250	0.026	−0.407	−0.026
Increase of R^2^ according to interaction terms:			R^2^-chnd		F	*p*
			0.019		5.062	0.026
Model 2 (outcome variable: professional achievement (PEF))						
Constant	3.066	0.375	8.180	0.000	2.323	3.808
RES → SEF	0.121	0.071	1.709	0.090	−0.019	0.261
VCC → SEF	0.340	0.090	3.765	0.000	0.161	0.519

(β), Unstandardized coefficient; SE, standard error; LLCI and ULCI, Bias-corrected 95% confidence interval of lower and upper limit.

Second, in the examination of the relationship between the resilience of the cadets and the professional achievement, it was found that the resilience of the cadets did not have a significant impact on the professional achievement (RES → SEF, for H2: β = 0.121, *p* = 0.090, see Model 2, Table 4). Consequently, hypothesis H2 was rejected.

Third, hypothesis H3 was formulated to explore the impact of self-efficacy on professional achievement, and the analysis demonstrated that self-efficacy has a positive and statistically significant effect on professional achievement (SEF → PEF, for H3: β = 0.340, *p* < 0.01, see Model 2, Table 4).

In the fourth stage of our analysis, the interaction between the resilience of the cadets and the vocational calling was observed to produce a statistically significant result (RES × VCC → SEF, for H5: β = − 0.217, *p* < 0.05). This significant finding aligns with hypothesis H5, which postulated the existence of a modulating effect within the model. The proportion of variance explained by this interaction, indicated as R2, was determined to be 0.019, with a significance level of p < 0.05, underscoring the empirical support for the hypothesized moderating influence (see Table 4). This outcome serves as a noteworthy validation of the intricate interplay between resilience, vocational calling, and their combined effect on the outcome variable, thereby advancing our understanding of this dynamic relationship.

Fifth, it was established that the perceived resilience of the cadets indeed validates the mediating role of self-efficacy in its association with professional achievement (see Table 5). The total effect within the pathway from the resilience of cadets to professional achievement was found to be β = 0.272 (*p* < 0.001), with a direct effect of β = 0.121 (*p* = 0.090). The supporting evidence for the indirect effect of self-efficacy as a mediator was confirmed through a bootstrapping analysis, as evidenced by the absence of zero within the confidence interval bounds. This observation supports hypothesis H4, which postulated that self-efficacy would act as a mediator factor in the relationship between cadets’ resilience and their professional achievement.

**Table 5:** The total, direct, and indirect effects of cadets’ resilience on professional achievements.

Resilience (RES)	Effect (β)	SE	LLCI	ULCI
Total effect	0.272	0.061	0.151	0.394
Direct effect	0.121	0.071	−0.019	0.261
Indirect effect	0.151	0.053	0.055	0.267

Effect (β), Unstandardized coefficient; SE, standard error; LLCI and ULCI, Bias-corrected 95% confidence interval of lower and upper limit.

The conditional effect of the resilience of the cadets in relation to the vocational calling was found to be significant when comparing the vocational calling values from M − 1SD (−0.435) to the mean (0.000), but not in the case of M + 1SD (0.435). Specifically, if the vocational calling was high, the effect of self-efficacy on professional achievement was observed to be not significant (see Table 6).

**Table 6:** The conditional effect of cadets’ resilience according to vocational calling.

Vocational calling (VCC)	Effect (β)	SE	*t*	*p*	LLCI	ULCI
−0.435 (M − 1SD)	0.283	0.059	40.833	0.000	0.167	0.399
0.000 (M)	0.189	0.059	30.227	0.002	0.073	0.305
0.435 (M + 1SD)	0.095	0.083	10.139	0.257	−0.070	0.260

M, mean; SD, standard deviation; Effect (β), Unstandardized coefficient; SE, standard error; LLCI and ULCI, Bias-corrected 95% confidence interval of lower and upper limit.

The region of significance, determined using the Johnson-Neyman method of illumination analysis for the complete range of moderating variables, is presented in Table 7. This approach provides a means to discern areas where the moderating effect based on the moderating variable is significant. The influence of cadet resilience on professional achievement through self-efficacy was found to be notable in regions where vocational calling values were below zero (‘0’).

**Table 7:** Conditional effect of focal predictor at values of the moderator.

Vocational calling (VCC)	Effect (β)	**SE**	*t*	*p*	LLCI	ULCI
−1.452	0.504	0.131	3.848	0.000	0.244	0.763
−1.342	0.480	0.121	3.954	0.000	0.239	0.720
−1.232	0.456	0.112	4.071	0.000	0.234	0.678
−1.122	0.432	0.103	4.200	0.000	0.228	0.636
−1.012	0.408	0.094	4.340	0.000	0.222	0.595
−0.902	0.384	0.086	4.487	0.000	0.215	0.554
−0.792	0.361	0.078	4.633	0.000	0.206	0.515
−0.682	0.337	0.071	4.764	0.000	0.197	0.477
−0.572	0.313	0.065	4.851	0.000	0.185	0.441
−0.462	0.289	0.060	4.852	0.000	0.171	0.407
−0.352	0.265	0.056	4.717	0.000	0.154	0.377
−0.242	0.242	0.055	4.408	0.000	0.133	0.350
−0.132	0.218	0.055	3.933	0.000	0.108	0.327
−0.022	0.194	0.058	3.350	0.001	0.079	0.308
0.088	0.170	0.062	2.737	0.007	0.047	0.293
0.198	0.146	0.068	2.157	0.033	0.012	0.280
0.234	0.138	0.070	1.980	0.050	0.000	0.277
0.308	0.122	0.075	1.642	0.103	−0.025	0.270
0.418	0.099	0.082	1.201	0.232	−0.064	0.261
0.528	0.075	0.090	0.828	0.409	−0.104	0.253
0.638	0.051	0.099	0.515	0.608	−0.145	0.247
0.748	0.027	0.108	0.251	0.802	−0.187	0.241

**Figure 2:**
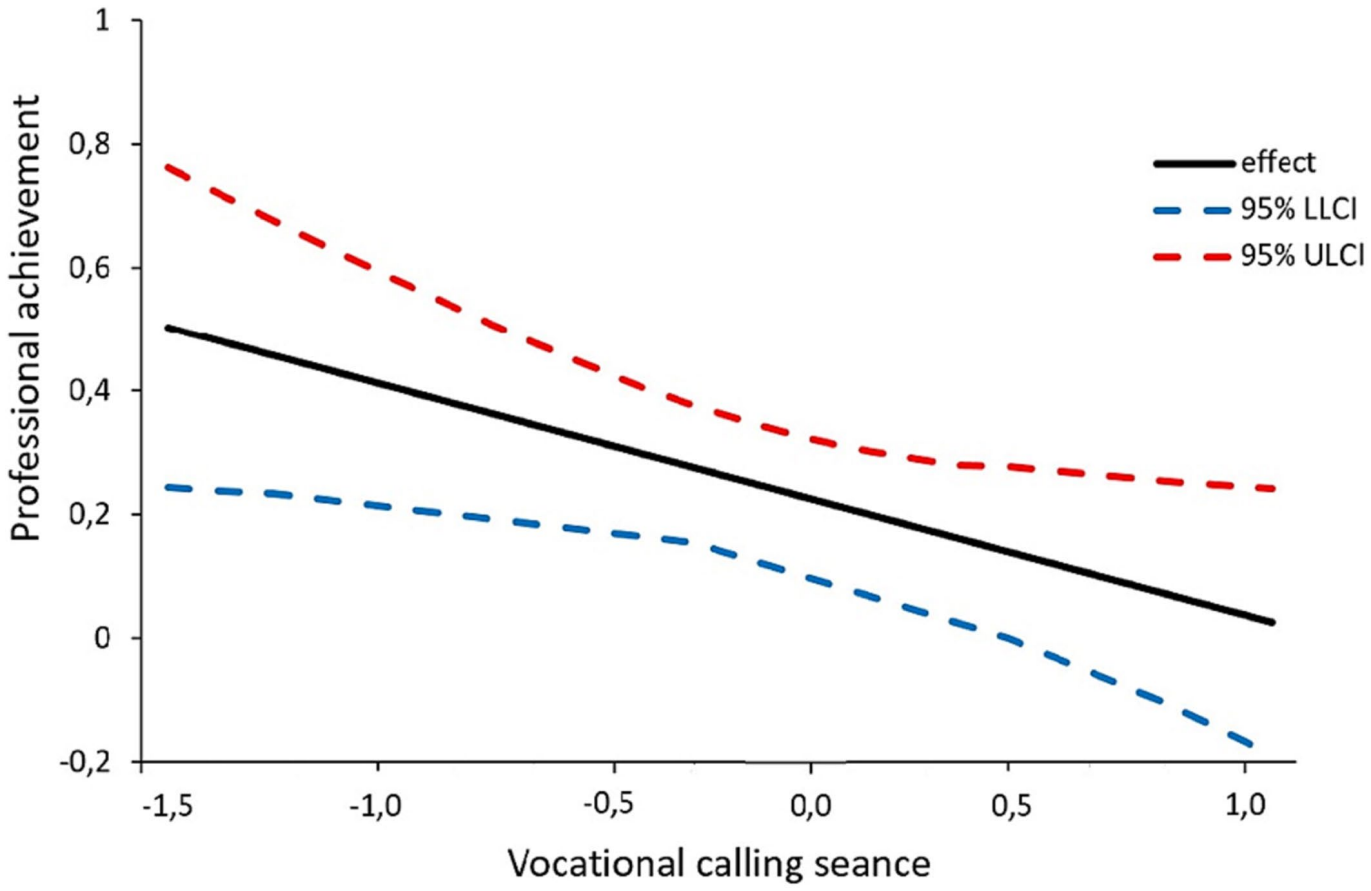
Graphical representation of the conditional effect of cadets’ resilience on self-efficacy at values of the moderator vocational calling.

In other words, in cases where the value of vocational calling was less than ‘0.000’, vocational calling played a significant role in moderating the mediating effect of self-efficacy in the relationship between the resilience of the cadets and professional achievement. Since the moderating impact of vocational calling was statistically significant, the results of this moderating effect were visualized in Figure 2 to illustrate the form of this interaction.

To understand the pattern of this significant interaction, vocational callings were categorized into low, medium, and high groups to examine average changes. It was observed that when self-efficacy was low, the group with higher vocational calling exhibited lower levels of professional achievement compared to the group with lower vocational calling. On the contrary, the group with lower vocational calling demonstrated higher professional achievement, even when self-efficacy was high.

The indirect conditional effect of vocational calling (VCC) on the relationships between cadets’ resilience (RES) and professional achievement (PEF) was found to be significant when comparing vocational calling values from M − 1SD (−0.435) to the mean (0.000), but it was not significant in the case of M + 1SD (0.435). Specifically, a low or average level of vocational calling was associated with a moderated mediating effect of vocational calling on the impact of cadets’ resilience on professional achievement through self-efficacy.

Furthermore, the moderated mediation index of vocational calling was calculated to be −0.074, and hypothesis H6, which postulates the existence of a moderated mediation effect, was supported with a 95% confidence interval (CI) that did not include zero in the lower limit (−0.180) and the upper limit (−0.015; see Table 8).

**Table 8:** The conditional effect taking into account the vocational calling.

Self-efficacy (SEF)	Effect (β)	SE	LLCI	ULCI
−0.435 (M − 1SD)	0.096	0.041	0.028	0.188
0.000 (M)	0.064	0.033	0.014	0.143
0.435 (M + 1SD)	0.032	0.039	−0.032	0.125
**Index of moderated mediation**		**SE**	**LLCI**	**ULCI**
−0.074		0.051	−0.180	−0.015

M, mean; SD, standard deviation; Effect (β), Unstandardized coefficient; SE, standard error; LLCI and ULCI, Bias-corrected 95% confidence interval of lower and upper limit.

## 6 Discussion

This research aimed to evaluate the direct and indirect relationships between resilience and personal achievements of the cadets. Our objective was to identify the key factors essential for the personal achievements of cadets as future officers and empirically analyse the conceptual framework that can guide the development of resources. In this context, we analysed a model of cadets’ resilience, examining the relationship between self-efficacy and personal professional achievements, and we also verified the moderated mediating effect of vocational calling.

First, vocational calling was found to positively affect the self-efficacy of participants under the resilience of the cadets. Future officers should be interdependent in situations that are not independent. Cadets resilience was found to affect their self-efficacy positively. Military resilience involves maintaining high levels of operational readiness even in the face of changing and unpredictable circumstances (Beckner et al., 2022; Bekesiene et al., 2022, 2023; Flood and Keegan, 2022). This readiness enables military forces to respond promptly and effectively to various threats, whether they are natural disasters or security challenges. Resilience in the military requires the ability to adapt to new and evolving threats, technologies, and tactics. Military organizations must continuously assess and adjust their strategies, equipment, and training to remain effective and resilient in a dynamic environment. Training plays a crucial role in building military resilience. Soldiers are physically and mentally prepared to endure hardships, respond to crises, and carry out their duties under adverse conditions. This training includes simulated exercises, scenario planning, and drills (Wampler et al., 2006; Mjelde et al., 2016; Kanapeckaitė et al., 2022; Bekesiene, 2023). Moreover, leaders within military organizations must exhibit resilience in their decision-making, adaptability to changing situations, and the ability to inspire and lead their troops effectively, especially in high-stress and uncertain environments (Jackson et al., 2012; Adler and Castro, 2013; Southwick et al., 2017).

Self-efficacy has also been shown to play a fully mediating role in the relationship between cadets’ resilience and professional achievements. In the dynamic and demanding world of military service, the journey from cadet to officer is marked by numerous challenges and transformations. Cadets, the future leaders of our armed forces, undergo rigorous training and face a constantly evolving environment. To excel in this role, they must not only possess the required skills but also a vital psychological attribute: self-efficacy. Self-efficacy, a concept introduced by renowned psychologist Bandura (1999, 2001, 2003, 2012), refers to an individual’s belief in their capacity to accomplish specific tasks and attain goals. It is a central component of social cognitive theory, emphasizing the interection between cognitive, behavioral, and environmental factors. In particular, the journey from cadet to officer is marked by moments of adversity and setbacks. Furthermore, high self-efficacy equips cadets with the mental resilience needed to bounce back from failures, learn from experiences, and persevere in the face of obstacles. In addition, self-efficacy serves as a powerful motivator, and cadets who believe in their abilities are intrinsically driven to excel (Boe et al., 2018). This internal motivation is more sustainable than extrinsic factors and fuels their determination to achieve professional excellence. It means that a cadet’s self-efficacy is a primary psychological mechanism for accepting change and professional achievement for military (Souza et al., 2014).

Therefore, it is meaningful to verify the statistical mediating effect of self-efficacy from the perspective of the cadets, and to try on their own for self-development with the support of their resilience. Vocational calling also plays a moderating role in the relationship between cadet resilience and self-efficacy and has a conditional effect on cadet resilience and professional achievements. The vocational calling of the study participants serves as a valuable source of guidance, allowing them to make prompt and informed decisions when faced with significant dilemmas. Nevertheless, the empirical findings of this study suggest that an excessive level of self-consciousness or an unwavering belief in one’s calling can impede professional achievements when not balanced with self-efficacy.

Lastly, this result diverges from previous studies (Lee, 2016; Afsar et al., 2019b), which suggested a positive impact of the sense of calling on work performance. Instead, our findings align with the conclusions drawn by Ji and Yoon (2021).

The research carried out has limitations that must be considered when interpreting the presented results. A general limitation is that the instrument used to assess the strengths of self-efficacy was based on self-evaluation and self-perception. Additionally, contextual factors related to the geopolitical situation during this study may have influenced the self-esteem of the cadets. It is also noteworthy that while self-efficacy beliefs and vocational calling can offer valuable insights into predicting cadets’ military performance, they are not the sole determinants. Military performance is a multifaceted outcome influenced by numerous factors, including training, experience, teamwork, leadership, and situational demands. Additionally, other variables such as aptitude, study behaviors, social support, and specifically environmental factors contribute to the overall outcomes observed in cadets.

## 7 Conclusion

In conclusion, the conducted study expands the understanding of the moderating role of vocational calling that has important implications for military training and cadet development. It highlights the importance of nurturing a sense of calling among cadets, as this can potentially enhance their resilience and self-efficacy. Also, can be noted that vocational calling is not only an inherent belief in the significance of one’s military service but also a sociopsychological variable that may have a moderating effect on the relationship between cadets’ resilience and self-efficacy. This hypothesis underscores the multifaceted nature of military success and the interplay between psychological attributes and a profound sense of purpose. It reinforces the importance of nurturing a vocational calling among cadets, as it can serve as a powerful catalyst for their resilience and self-efficacy, ultimately shaping their achievements within the military. Subsequent investigations within this domain should undertake a comprehensive exploration of the underlying mechanisms by which vocational calling exerts its influence on the psychological resources of cadets, thereby elucidating its ramifications on their aptitude to excel within the military sphere.

Further research efforts may encompass a multidimensional analysis of the intricate interplay between vocational calling, self-efficacy, and other pertinent psychological determinants to provide a more profound understanding of the dynamics at play. Based on the findings of this study, the following recommendations for future research can be presented: (1) expand the sample size to further validate the structural relationships between variables; (2) conduct follow-up studies to analyse differences between cadet groups based on study year, gender, and military service experience; (3) additionally, it can be consider introducing leadership styles and organizational culture as predictors of organizational psychology.

## Data availability statement

The raw data supporting the conclusions of this article will be made available by the authors, without undue reservation.

## Ethics statement

The studies involving humans were approved by General Jonas Žemaitis Military Academy of Lithuania. Written informed consent from the patients/participants or patients/participants' legal guardian/next of kin was not required to participate in this study in accordance with the national legislation and the institutional requirements.

## Author contributions

ON: Conceptualization, Data curation, Formal analysis, Funding acquisition, Investigation, Methodology, Project administration, Resources, Software, Supervision, Validation, Visualization, Writing – original draft, Writing – review & editing. AV: Conceptualization, Formal analysis, Funding acquisition, Investigation, Methodology, Project administration, Resources, Supervision, Validation, Visualization, Writing – original draft, Writing – review & editing.
